# Interlayer Exciton–Phonon Coupling in MoSe_2_/WSe_2_ Heterostructures

**DOI:** 10.1021/acs.nanolett.4c02757

**Published:** 2024-09-12

**Authors:** Oisín Garrity, Thomas Brumme, Annika Bergmann, Tobias Korn, Patryk Kusch, Stephanie Reich

**Affiliations:** † Department of Physics, Freie Universität Berlin, Arnimallee 14, D-14195 Berlin, Germany; ‡ Chair of Theoretical Chemistry, Technische Universität Dresden, Bergstraße 66, 01069 Dresden, Germany; § Institute of Physics, Universität Rostock, 18059 Rostock, Germany

**Keywords:** exciton−phonon, Raman, TMDC, DFT, heterostructures

## Abstract

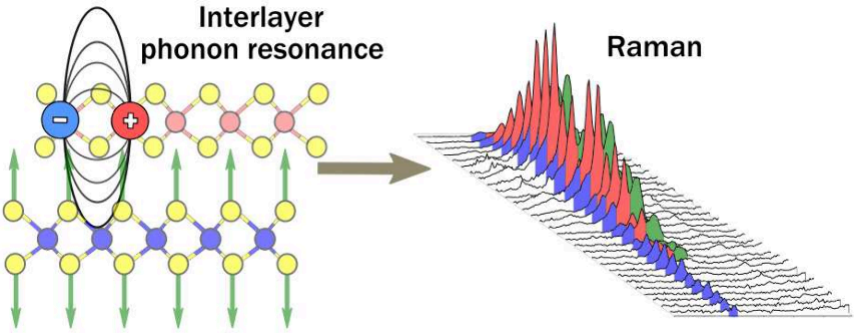

Transition metal dichalcogenide heterostructures have garnered strong interest for their robust excitonic properties, mixed light–matter states such as exciton-polaritons, and tailored properties, vital for advanced device engineering. Two-dimensional heterostructures inherit their physics from monolayers with the addition of interlayer processes that have been particularly emphasized for their electronic and optical properties. Here, we demonstrate the interlayer coupling of the MoSe_2_ phonons to WSe_2_ excitons in a WSe_2_/MoSe_2_ heterostructure using resonant Raman scattering. The WSe_2_ monolayer induces an interlayer resonance in the Raman cross-section of the MoSe_2_ A_1g_ phonons. Frozen-phonon calculations within density functional theory reveal a strong deformation-potential coupling between the A_1g_ MoSe_2_ phonon and the electronic states of the close-by WSe_2_ layer approaching 20% of the intralayer coupling to the MoSe_2_ electrons. Understanding the vibrational properties of van der Waals heterostructures requires going beyond the sum of their constituents and considering cross-material coupling.

Van der Waals heterostructures combine monolayer two-dimensional materials into stacks with atomic precision, which achieves an intimate connection of widely different materials.^1^ In such stacks, transitional metal dichalcogenides (TMDCs) are of particular interest due to their optical properties originating from excitons with large binding energies having potential applications in valleytronics and as hybridized light–matter states.^2−6^ While van-der-Waals heterostructures were first seen as mere combinations of disparate materials, researchers soon realized that their most intriguing properties arise from a coupling of quasiparticles in different layers. This interlayer interaction induce, e.g., superconductivity in twisted graphene bilayers and interlayer excitons from charge carriers situated in distinct layers.^7,8^

In contrast to optical and electronic properties, the vibrational response of heterostructures (with the exception of interlayer shear and breathing modes^9^) has been mainly perceived as the sum of the heterostructure components. Unravelling interlayer vibrational interaction is particularly important when considering that phonons provide the prime relaxation channels for excited charge carriers, are involved in heat transport, and determine exciton dynamics.^10,11^ A first indication for nontrivial effects of van der Waals stacking on phonons was the appearance of silent h-BN phonons in the Raman spectrum of a WSe_2_/h-BN heterostructure. It was attributed to an interaction of the h-BN phonons with a new optical transition of the heterostructure.^12^ Other indications of interlayer exciton–phonon coupling were twist-induced scattering for the double-resonant Raman process of graphene as well as a preserved photohelicity in the emission from an h-BN/WSe_2_/h-BN heterostructure.^13,14^ Reports of interlayer vibrational coupling to date have focused on the activation of new Raman modes by breaking the point and translational symmetries. No study has followed exciton–phonon coupling in TMD heterostructures across their excitonic resonance to observe the coupling between Raman-allowed phonons and the optical excitations of such devices. We need to unravel the vibrational dimension of the interlayer interaction to fully understand and engineer the heterostructure response.

In this paper, we directly observe interlayer exciton–phonon coupling in a WSe_2_/MoSe_2_ heterostructure using multiwavelength Raman scattering in resonance with the A excitons of the two materials (1.5–1.8 eV). We observe a WSe_2_-related Raman resonance for the A_1g_ mode of MoSe_2_, meaning that the A_1g_ phonon of MoSe_2_ scatters with the A exciton in the WSe_2_ layer (interlayer) in addition to its scattering with the A exciton of MoSe_2_ (intralayer). In contrast, no interlayer resonance is observed for the WSe_2_ A_1g_ phonon. The inter- and intralayer resonances of the A_1g_ phonons are explained by the strength of light–matter interaction and exciton–phonon coupling in the two materials, as we show by ab initio calculations of the phonon-deformation potential. The intralayer interactions open additional relaxation channels for excited carriers in TMD heterostructures compared to the pristine material. Our findings also imply that Raman characterization of van der Waals materials needs to consider interlayer coupling.

We first discuss the electronic states and optical transitions of the MoSe_2_/WSe_2_ heterostructure. In an MoSe_2_/WSe_2_ heterostructure with strong layer interaction, the electronic band configuration is a type II staggered alignment, meaning the conduction band minimum is localized in the MoSe_2_ layer while the valence band maximum is localized in the WSe_2_ layer as illustrated in Figure 1.^15,16^ The blue label XA^M^ indicates the transitions that lead to the A exciton at the *K* point for MoSe_2_ and the red label XA^*W*^ for WSe_2_ monolayers. These momentum-direct excitons have a transition energy of 1.59 eV in MoSe_2_ (blue) and 1.66 eV in WSe_2_ (red) for the monolayer case, and 1.57 eV in MoSe_2_ and 1.64 eV in WSe_2_ for the heterostructure case (black), see PL in Figure 2b.^15^ The label XI denotes interlayer excitons formed by combining the electronic states of MoSe_2_ and WSe_2_.^17^

**Figure 1:**
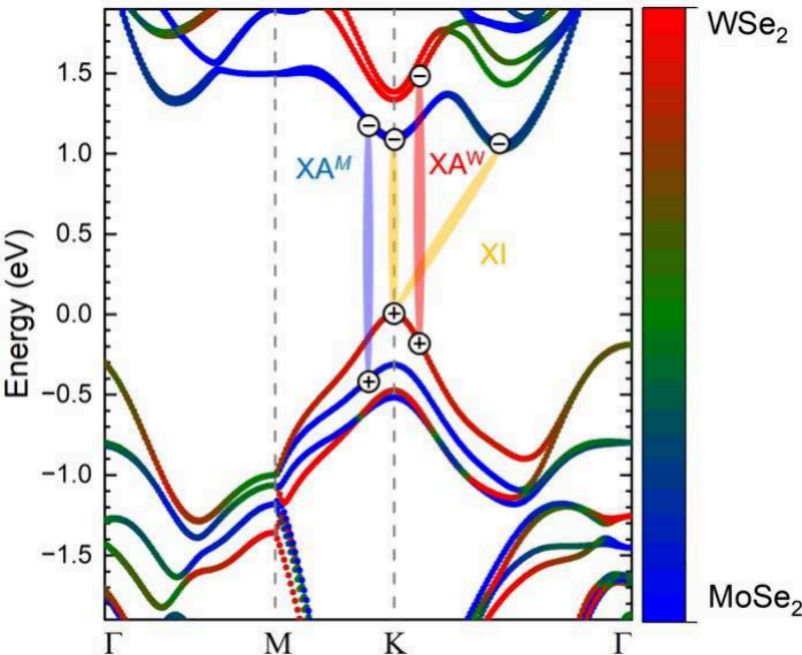
Electronic dispersion of the MoSe_2_/WSe_2_ heterostructure showing a staggered band alignment. The color scale indicates in which layer the bands are localized, blue for MoSe_2_, red for WSe_2_, and green for an equal band contribution from both layers. The blue label XA^*M*^ refers to the A-excitonic transition in MoSe_2_, while the red label XA^*W*^ is the A-excitonic transition in WSe_2_. The interlayer transition, labeled XI, originates from combining MoSe_2_ and WSe_2_ states into an exciton.

**Figure 2:**
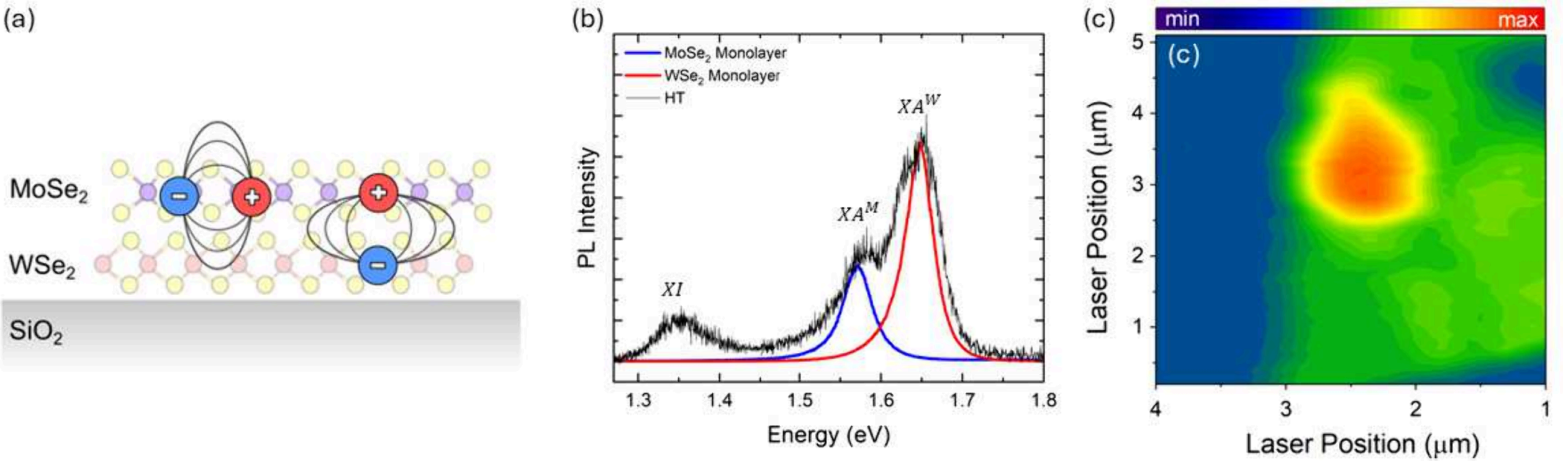
(a) Sketch of an MoSe_2_/WSe_2_ heterostructure on a SiO_2_. Featured are an intralayer exciton (left) with electron and hole within one layer and an interlayer exciton (right) where the bound electron and hole are localized in different layers. (b) Room-temperature PL spectra of monolayer MoSe_2_ (blue, scaled down), monolayer WSe_2_ (red, scaled down), and the heterostructure (black) from intralayer A-exciton transitions of 1.57 eV (MoSe_2_) and 1.64 eV (WSe_2_) and the interlayer exciton transition of 1.34 eV. (c) PL intensity map taken at 1.35 eV at room temperature.

When the layers are decoupled or interact only weakly, the heterostructure emits light from the intralayer XA excitons, Figure 2a, of both layers, as seen at 1.57 eV (MoSe_2_) and 1.66 eV (WSe_2_) in the spectrum in Figure 2b. For strongly interacting layers, some charge carriers participate in the interlayer XI exciton emission at 1.35 eV, lowering the intensity of the XA excitons, black line in Figure 2b.^15,17,18^ The peak energy and relative intensities of the interlayer PL in our device suggest an H-type stacking of the two layers, i.e., a twist angle of ∼60°.^19^ A map of the interlayer PL intensity in Figure 2c shows areas of strong interaction (red), individual WSe_2_ monolayer (green), and the SiO_2_ substrate (blue); this was used to identify individual layers and strongly coupled areas of the MoSe_2_/WSe_2_ heterostructures for the Raman experiments.

Figure 3 shows Raman spectra taken with tunable laser excitations in the spectral region of the XA excitons of (a) a MoSe_2_ monolayer with the out-of-plane phonon mode A_1g_^*M*^ at 242 cm^–1^ (blue arrow) and (b) a WSe_2_ monolayer with the out-of-plane phonon mode A_1g_^*W*^ at 250 cm^–1^ (red arrow) and the peak by the overtone of the longitudinal acoustic phonon 2LA at 261 cm^–1^ (gray arrow).^20−22^ These three Raman peaks also appear in the MoSe_2_/WSe_2_ heterostructure, Figure 3c, but for a given wavelength with somewhat altered intensities compared to those in the monolayer case. This change in the relative scattering intensities is caused by changes in the electronic structure and electron–phonon coupling due to the stacking.^23^ The quantum theory of the Raman effect describes it as a three-step process of (i) absorption of an incoming photon and creation of an electron–hole pair, (ii) scattering of the carriers under phonon emission via electron–phonon scattering, and (iii) recombination of the electron–hole pair at different energies. The energy difference between the incoming and scattered light is given by the phonon energy, but the scattering intensity is given by light–matter (steps i and iii) and electron–phonon coupling, (step ii). When varying the laser energy, Figure 3, the scattering intensities of the A_1g_^*M*^ mode (blue arrow) and the A_1g_^*W*^ mode (red arrow) vary compared to the constant scattering by Si.^24^ The integrated intensity of a Raman peak greatly increases when the laser energy matches a real electronic transition in a material such as the XA excitons. This reflects the increasing probability for photon absorption and emission (steps i and iii) and allows the study of electronic transitions and their coupling to phonons through the Raman effect. No Raman peaks were observed from the heterostructure, with laser energies at the XI exciton (1.35 eV). The XI transition induced no resonance in the Raman cross sections because it originates from an indirect excitation via XA states.

**Figure 3:**
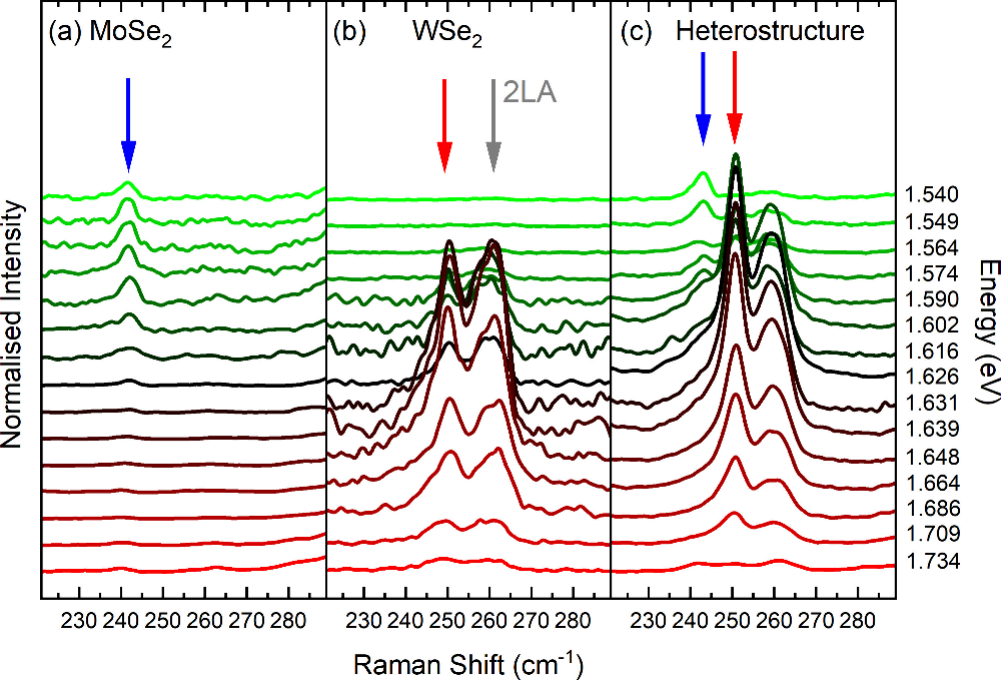
Raman spectra excited with tunable laser wavelength/energy at 1.5–1.8 eV (see labels) of the (a) MoSe_2_ mode A_1g_^*M*^ at 242 cm^–1^ (blue arrow), (b) WSe_2_ mode A_1g_^*W*^ at 250 cm^–1^ (red arrow) and 2LA mode at 261 cm^–1^ (gray), and (c) heterostructure A_1g_ modes from MoSe_2_ (blue arrow) and WSe_2_ (red arrow). All spectra measured at room temperature under ambient conditions and normalized to the Si scattering intensity.

To quantitatively analyze the A_1g_ resonances, we determined the integrated intensity peaks in Figure 3a–c by fitting the spectra and extracting the area under the curve, see the Supporting Information. From the fits, we prepared a resonance Raman profile, shown in Figure 4, i.e., a plot of the integrated peak intensity as a function of excitation energy. The resonance profile is described within the quantum theory of Raman scattering discussed above using third-order perturbation theory,^23,25^
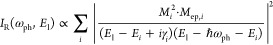
1In this expression, *M*_*i*_ is the electron-photon matrix element of the electronic transition *i* and *M*_ep,*i*_ the electron–phonon matrix element of the excited charge carrier. *E*_l_ is the excitation laser energy. ω_ph_ is the phonon frequency, and γ_*i*_ is the decay rate of the excited electronic state. We simplified the most general expression for the Raman cross section by introducing distinct intermediate excitonic states *E*_*i*_ (*i* represents the XA exciton of MoSe_2_ or WSe_2_).^25^ When the laser energy approaches a material transition energy, *E*_l_ ≈ *E*_*i*_ in eq 1 and the real part of the denominator vanishes, giving rise to a Raman resonance. Note that each transition induces two resonances, an incoming resonance if the laser matches an electronic transition and an outgoing resonance if the scattered photon matches the transition energy. The electron–photon and electron–phonon matrix elements in eq 1 are considered to be constant and are typically treated as one fitting parameter in the analysis of resonance profiles. The measured Raman profiles we fit with *I*_R_, full lines in Figure 4; the parameters are given in Table 1.

**Figure 4:**
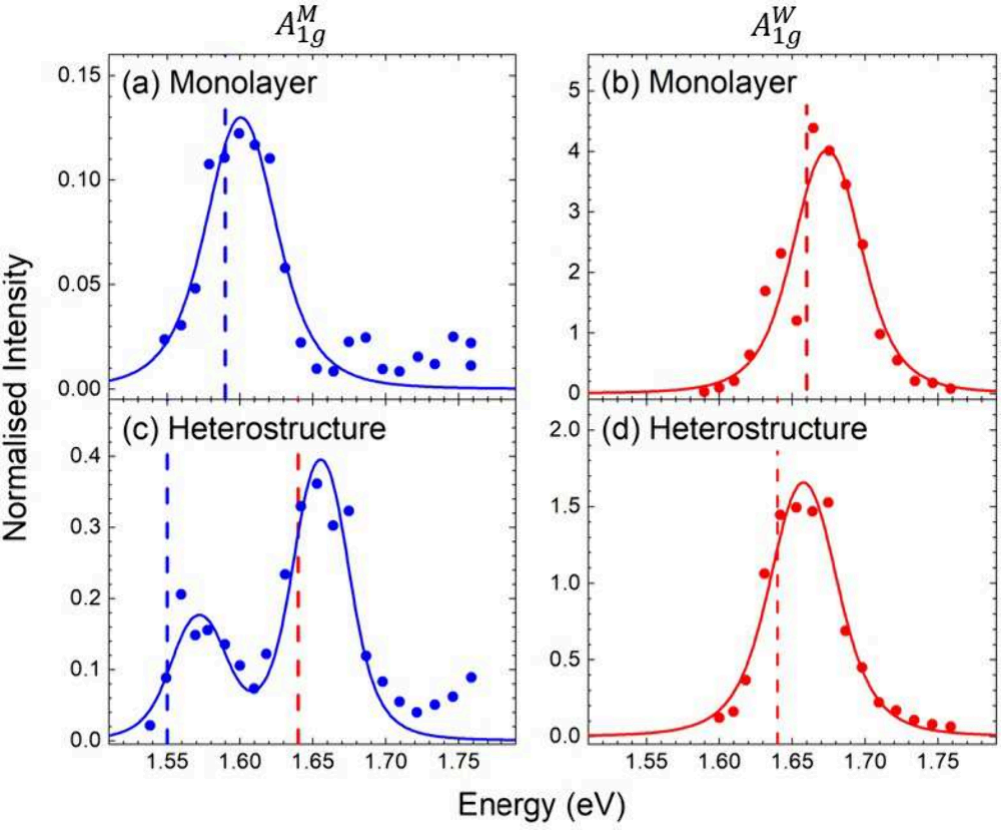
Resonant Raman profiles of the heterostructure and their monolayers. Data points are room-temperature integrated peak intensities normalized to Si of the (a) A_1g_^*M*^ monolayer, (b) A_1g_^*W*^ monolayer, (c) A_1g_^*M*^ heterostructure, and (d) A_1g_^*W*^ heterostructure profiles. Full lines are fits with eq 1, see Table 1 and Supporting Information. Dashed lines mark the fitted energies of the XA excitons originting from the blue MoSe_2_ and red WSe_2_ layer.

**Table 1:** Fits of the Raman Resonance Profiles with eq 1 Giving the A_1g_ Frequency, the XA Resonance Energy, and the Decay Rate γ^a^

		monolayer		heterostructure	
	A_1g_ (cm^–1^)	*X*_A_ (eV)	γ_r_ (eV)	*X*_A_ (eV)	γ_r_ (eV)
MoSe_2_	242	1.59	0.08	1.55	0.05
				1.64	0.05
WSe_2_	250	1.66	0.06	1.64	0.06

a The MoSe_2_ of the heterostructure shows a second resonance at the energy of the XA^*W*^ exciton.

The A_1g_ Raman profiles show resonances from intralayer coupling to the XA excitons of the monolayers or heterostructure components, as shown in Figure 4. These resonances shift by up to 40 meV to smaller energies in the MoSe_2_/WSe_2_ heterostructure, Table 1. Similar shifts have been noted before in TMD heterostructures and attributed to strain, doping, and the change in dielectric environment due to the second layer.^26−28^ The most intriguing feature in the resonance profiles of the MoSe_2_/WSe_2_ heterostructure, however, is the additional second resonance of the MoSe_2_ derived A_1g_^*M*^ phonon at the XA exciton energy of WSe_2_. This manifests an interlayer exciton–phonon coupling in the heterostructure. The interlayer resonance is stronger in intensity than the intralayer resonance, which appears counterintuitive and requires further examination.

When the three steps in the Raman process are considered, only electron–phonon coupling can give rise to an interlayer Raman resonance. A photon that matches the XA^*M*^ cannot create an exciton in the WSe_2_ component at energy XA^*W*^ and vice versa, ruling out an exciton-induced interlayer coupling. On the other hand, there are two mechanisms for the interlayer interaction in the phonon part of the Raman process: The phonons of the monolayer may mix in the heterostructure, so that a predominantly A_1g_^*M*^ phonon of MoSe_2_ also leads to vibrations in the WSe_2_ layer. Alternatively, the electron wave functions of one monolayer may extend into the other layer and get affected by the vibration of the nearby atoms. To understand the interlayer electron–phonon coupling in the MoSe_2_/WSe_2_ heterostructure, we calculated the phonon frequencies and eigenvectors as well as their electron–phonon coupling using density functional theory.

Indeed, the eigenvectors of the heterostructure mix between the two layers, as shown in Figure 5a. The A_1g_^*M*^ mode of the heterostructure that is predominantly an out-of-plane vibration of the MoSe_2_ selenide atoms, also involves a movement of the WSe_2_ atoms and vice versa. In addition, the symmetry breaking by the heterostructure induces movement of both chalcogen atoms vibrating against each other. The eigenfrequencies of the heterostructure are 1 cm^–1^ higher than in the monolayer. The mixing of the eigenvectors results in an induced movement in the adjacent layer that is as strong as 17% of the source layers movement, see Supporting Information Table 2.

**Figure 5:**
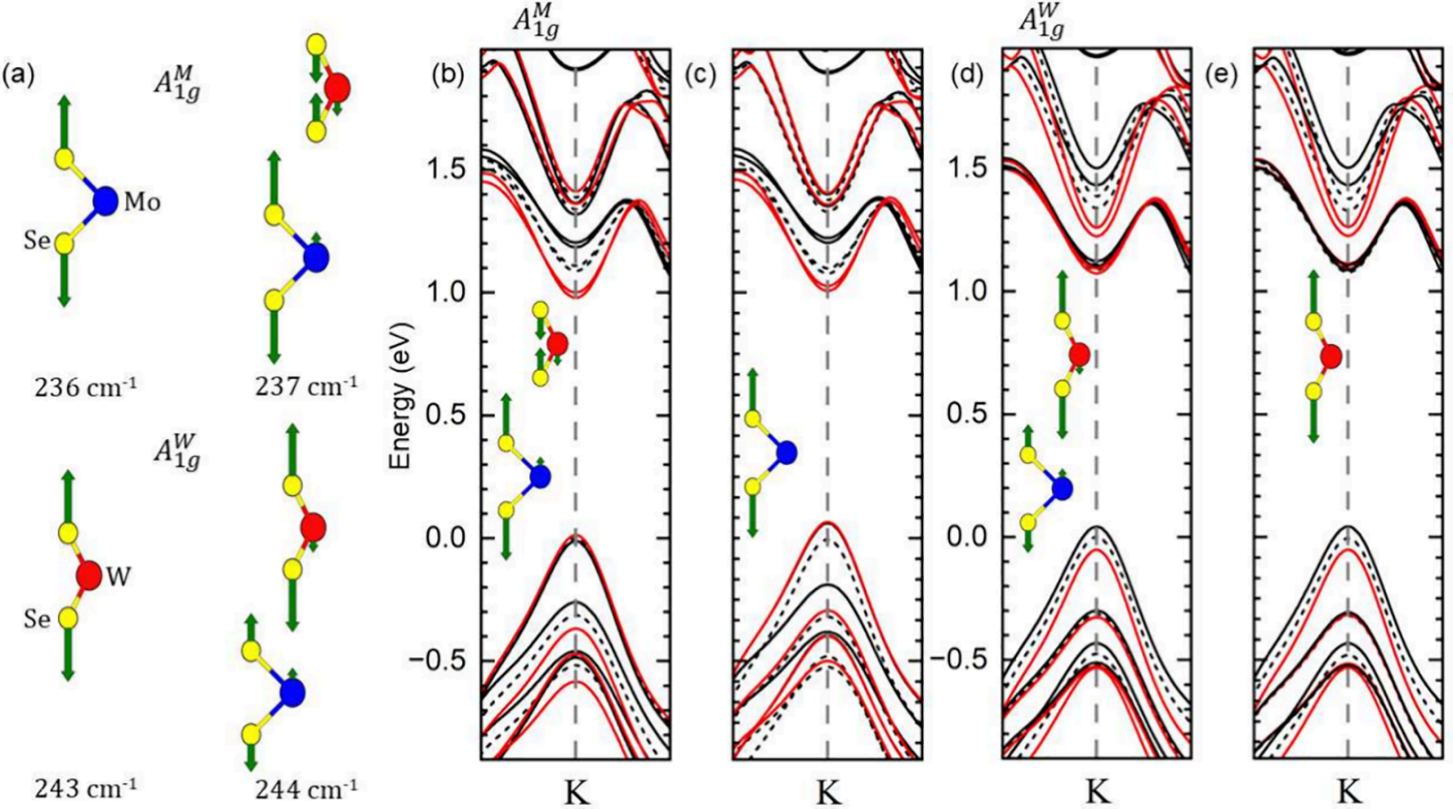
(a) Eigenvectors and frequencies of the A_1g_ modes for the monolayers (left) and the heterostructure (right). The displacement vectors of the induced vibrations are magnified for clarity; for the unaltered vectors, see Supporting Information Table 2. (b–d) Change of the electronic states in the heterostructure under an atomic displacement according to the eigenvectors in the inset. Red lines are for positive, black lines for negative displacement, and dashed lines for the equilibrium atomic positions. (b) Eigenvector A_1g_^*M*^ in a and c monolayer eigenvector with the WSe_2_ atoms fixed at equilibrium position; c and d are the corresponding plots for the A_1g_^*W*^ mode.

The deformation potential simulation finds both intra- and interlayer electron–phonon coupling for the A_1g_ modes of the heterostructure. The intralayer deformation potentials for H-type stacking is 104 eV/Å for A_1g_^*M*^/XA^*M*^ coupling—the intralayer coupling of the MoSe_2_A_1g_^*M*^ mode to the MoSe_2_ electrons—and 194 eV/Å for A_1g_^*W*^/XA^*W*^ coupling. This is comparable to electron–phonon coupling in graphene^29^ but larger than in classical semiconductors such as GaAs.^30^ The calculations predict the interlayer coupling of the A_1g_^*M*^ mode to the XA^*W*^ exciton A_1g_^*M*^/XA^*W*^ (22 eV/Å) to be 20% of the intralayer coupling. The absolute numbers in Table 2 differ somewhat for R-type stacking, but overall, our calculations find interlayer electron–phonon interaction in the MoSe_2_/WSe_2_ heterostructure, in excellent agreement with experimental results.

**Table 2:** Deformation Potentials for the A_1g_^*M*^ and A_1g_^*W*^ Heterostructure Eigenvectors on the MoSe_2_ and WSe_2_ Bands at *K* (○ = *M*, *W*)^a^

	*M*_A_1g_^*M*^/XA°_ (eV/Å)	*M*_A_1g_^*W*^/XA°_ (eV/Å)	ratio
H-type stacking			
*M*_*A*_1g_°/*XA*^*M*^_	104	22	0.21
*M*_*A*_1g_°/*XA*^*W*^_	4	194	0.02
R-type stacking			
*M*_*A*_1g_°/*XA*^*M*^_	160	28	0.18
*M*_*A*_1g_°/*XA*^*W*^_	17	199	0.09

a The other heterostructure stackings are reported in Supporting Information Table I.

To quantify if the larger contribution to the interlayer coupling is from the mixing of phonon eigenmodes, Figure 5a, or from the wave function overlap with the adjacent layer, we repeated the deformation potential calculations using the monolayer phonon eigenvector for one layer keeping the other layer fixed in position. The resulting bandstructures around *K* differ little between the simulations of the hybridized, Figure 5b and d, and the monolayer eigenvectors, Figure 5c and e. The major contributor to the interlayer electron–phonon coupling arises from the electronic states of the intralayer exciton overlapping with the adjacent layer so that the moving MoSe_2_ atoms affect the eigenenergies of the WSe_2_ electronic states. The mixing of the eigenvectors is of minor importance to interlayer coupling.

We now return to the surprisingly intense interlayer resonance of the A_1g_^*M*^ mode, Figure 4c. It originates from the strong absorption of XA^*W*^ compared to XA^*M*^ that counteracts the weaker interlayer coupling compared to that of the intralayer interaction. To show this, we consider the expression for the resonant Raman intensity eq 1 and approximate the transitions to intermediate excitonic states by the absorption coefficient α.^25^ Making use of ℏω_ph_ ≪ *E*_l_ for eq 1, we obtain
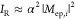
2The absorption coefficient for MoSe_2_ in the investigated spectral range is α^*M*^ ≈ 10^4^ cm^–1^ in contrast to α^*W*^ ≈ 10^5^ cm^–1^ for WSe_2_.^31,32^ The different exciton transition probabilities are also apparent from the monolayer PL intensities (see Supporting Information Figure 3), with an intensity ratio between the WSe_2_ and MoSe_2_:
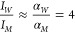
3To calculate the ratio between the interlayer and the intralayer scattering of A_1g_^*M*^ mode, we use the deformation potentials in Table 2 and obtain
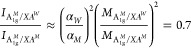
4Given the uncertainties in our estimates, this is in reasonably good agreement with the similar intensity of the interlayer (A_1g_^*M*^/XA^*W*^) and intralayer (A_1g_^*M*^/XA^*M*^) resonances in Figure 4c. For the A_1g_^*W*^ mode, in contrast, the ratio between the absorption coefficient is inverse, and the interlayer electron–phonon coupling is less than 10% of the intralayer coupling. This leads to an expected intensity ratio of 10^–5^ between the inter- and intralayer WSe_2_ phonon resonances. The interlayer A_1g_^*W*^ resonance is too weak to be detected in the heterostructure, in agreement with our experimental findings. Equation 2 also explains the different Raman intensities observed between the two monolayers. The intralayer coupling and the absorption coefficient are larger in the WSe_2_ than in the MoSe_2_ layer, yielding an estimated intensity ratio of 50 based on eq 2 compared to an experimental ratio of 30, Figure 4a and b.

In summary, we studied interlayer and intralayer exciton–phonon coupling in a MoSe_2_/WSe_2_ heterostructure using resonant Raman scattering and *ab initio* calculations. We find that excitons in the heterostructure interact with phonons in adjacent layers via the deformation potential mechanism. Experimentally, we observed that the intensity of the interlayer Raman resonance for the A_1g_ phonon of MoSe_2_ is comparable to that of the intralayer resonance within the material. This originates from the absorption coefficient of WSe_2_ and the relatively strong interlayer coupling of the MoSe_2_ phonon. We show, using density functional analysis, that the largest contribution to this interlayer exciton–phonon coupling is from the overlapping of the electronic states of the XA^*W*^ intralayer exciton with the MoSe_2_ layer, rather than the mixing of the A_1g_ phonon eigenvectors. In contrast, only intralayer resonances were observed in WSe_2_ due to the small interlayer exciton–phonon coupling of this mode and the weaker absorption in MoSe_2_. Understanding the vibrational properties of TMD heterostructures as well as their carrier relaxation requires the consideration of interlayer interactions beyond the emergence of interlayer excitons. The interlayer vibrational coupling will be beneficial for further device engineering. One could selectively create a resonance in a TMDC monolayer with the appropriate choice of crystal, with tailored electronic and vibrational properties, and with the appropriate phonon mode symmetry.

## Supporting Information Available

Experimental details and sample production as well as a description of the fitting procedure using eq 1 to fit the Raman spectra shown in Figure 3; additional figures showing the sample area, PL spectra from the monolayers, and a table of optical deformation potentials as well as how they were calculated (PDF)
