# Correction to “Interlayer Exciton–Phonon Coupling in MoSe_2_/WSe_2_ Heterostructures”

**DOI:** 10.1021/acs.nanolett.4c05914

**Published:** 2024-12-16

**Authors:** Oisín Garrity, Thomas Brumme, Annika Bergmann, Tobias Korn, Patryk Kusch, Stephanie Reich

Corrections to Table 2 in the original article are as follows.

**Table 2:** Deformation Potentials for the A_1*g*_^*M*^ and A_1*g*_^*W*^ Heterostructure Eigenvectors on the MoSe_2_ and WSe_2_ Bands at *K* (° = *M*, *W*)^a^

	*M*_*A*_1*g*_^M^/*XA*^°^_ (eV/Å)	*M*_*A*_1*g*_^°^/*XA*^*W*^_ (eV/Å)	ratio
H-type Stacking			
*M*_*A*_1*g*_^*M*^/*XA*°_	104	22	0.21
*M*_*A*_1*g*_^*W*^/*XA*°_	4	194	0.02
R-type Stacking			
*M*_*A*_1*g*_^*M*^/*XA*°_	160	28	0.18
*M*_*A*_1*g*_^*W*°^/*XA*°_	17	199	0.09

a The other heterostructure stackings are reported in Supporting Information Table I.

## Supporting Information Available

Experimental details and sample production as well as a description of the fitting procedure using eq 1 to fit the Raman spectra shown in Figure 3; additional figures showing the sample area, PL spectra from the monolayers, and a table of optical deformation potentials as well as how they were calculate (PDF)

