# Supplementary material for: Interlayer Exciton–Phonon Coupling in MoSe2/WSe2 Heterostructures
Source: Nano Lett. 2024 Sep 12;24(38):11853–8. doi: 10.1021/acs.nanolett.4c02757 (PMC11440638; doi:10.1021/acs.nanolett.4c02757)
Supplement: Supplementary file 1 — nl4c02757_si_001.pdf [file nl4c02757_si_001.pdf]

Interlayer Exciton-Phonon Coupling in $MoSe_2$/WSe_2 Heterostructures: Supplementary Material

Oisín Garrity[a],[1] Thomas Brumme[b],[2] Annika Bergmann[c],[3]
Tobias Korn[d],[3] Patryk Kusch[e],[1] and *Stephanie Reich[f1]

[1] *Department of Physics, Freie Universität Berlin, Arnimallee 14, D-14195 Berlin, Germany*

[2] *Chair of Theoretical Chemistry, Technische Universität Dresden, Bergstraße 66, 01069 Dresden, Germany*

[3] *Institute of Physics, Universität Rostock, 18059 Rostock, Germany*

(Dated: June 2024)

[a] Oisín Garrity - o.garrity@fu-berlin.de

[b] Thomas Brumme - tbrumme@msx.tu-dresden.de

[c] Annika Bergmann - annika.bergmann@uni-rostock.de

[d] Tobias Korn - tobias.korn@uni-rostock.de

[e] Patryk Kusch - patryk.kusch@fu-berlin.de

[f] Corresponding author: Stephanie Reich -reich@physik.fu-berlin.de

EXPERIMENT DETAILS

Sample production and Raman spectroscopy

$MoSe_2/WSe_2$ heterostructures were prepared via dry viscoelastic stamping [1]. $MoSe_2$ and WSe_2 monolayer flakes were mechanically exfoliated onto polydimethylsiloxane (PDMS). Such monolayers often accrue carbonaceous contaminants between the layer and the substrate making the fabrication of heterostructures with strong interlayer coupling and the right twist angle challenging [2–4]. We transferred the WSe_2 monolayer onto a SiO_2 substrate and deposited the $MoSe_2$ monolayer on top using deterministic transfer observed in-situ by optical microscopy. The crystallographic axis was identified from a straight edge the flakes to achieve either zero or sixty degrees of twist angle. The samples, contained in a test tube, were annealed in an oil bath at 400 K and 10^{-4} mbar pressure. This was to get rid of air bubbles and contaminants the layers and improve the interlayer coupling. Room temperature photoluminescence (PL) was excited with a 532nm laser (1 mW power) and detected by a Horiba XploRA spectrometer with an 0.90 NA 100x objective (NA is the numerical aperture).
Resonant Raman measurements were excited with a fully tuneable Ti:sapphire laser pumped by a Sprout-G 18W Nd:Yag laser. All measurements were performed at room temperature under ambient conditions. The excitation laser with a power of 1 mW was focused on the samples by an 0.8NA 100x objective, with spectra taken at 5 nm intervals from 700–800 nm ($1.5 - 1.8$ eV). The backscattered light was collected by the same objective and guided into a Horiba t64000 spectrometer in triple-grating configuration. The spectrometer was equipped with $900\,\mathrm{mm}{-}^{1}$ gratings and a silicon charge-coupled device (CCD) detector. The Raman spectra were were normalised to the constant Raman cross section of the Si peak at $521\,\mathrm{cm}^{-1}$ and fitted by Lorentzians with the intensities taken as the area under the curve. The normalised area was plotted as a function of excitation energy to obtain the Raman resonance profiles, see Supplementary Information for details.

Density functional theory

To calculate the electronic properties and electron-phonon interaction in the $MoSe_2/WSe_2$ heterobilayer, we utilised density functional theory (DFT) as implemented in the Quan-

tum ESPRESSO package which uses a plane-wave basis set to describe the valence-electron wave function and charge density [5, 6]. We employed full-relativistic, projector-augmented wave potentials [7] of version 1.0.0 of the pslibrary [8] except for W where we used a slightly modified version to allow for better convergence during the relaxation. We chose the Perdew-Burke-Ernzerhof functional [9] for the exchange-correlation energy and furthermore included dispersion corrections [10]. The cut-off for the wave functions and the charge density was 60 Ry and 600 Ry (1 Ry $\approx$ 13.6 eV) respectively. The Brillouin zone integration was performed with a Γ-centered Monkhorst-Pack grid [11] of 25 $\times$ 25 $\times$ 1 k points. The self-consistent solution of the Kohn-Sham equations was obtained when the total energy changed by less than 10^{-8} Ry and the maximum force on all atoms was less than 0.001 Ry a_0^{-1} ($a_0 \approx 0.529$ Å is the Bohr radius). The unit cells were relaxed to a stress of $<$ 0.25 kbar. To properly treat the 2D boundary conditions of the heterostructure, we used the Coulomb cut-off technique [12] as implemented in Quantum ESPRESSO. The phonons at the Γ point were calculated with the finite-displacement method as implemented in phonopy [13, 14] using a single unit cell. We verified that the phonon energies as well as the eigenvectors (at Γ) are not influenced by the restriction to a single unit cell by also calculating the phonon dispersion for a 5 $\times$ 5 $\times$ 1 supercell for one specific stacking.

To obtain the electron-phonon coupling with deformation potential theory [15–17], Table I, we evaluated the electron-phonon matrix element

$$M_{ep,i} = \langle km| \, H^i_{ep} \, |m'k\rangle = \sum_a \sqrt{\frac{\hbar}{2M_a N_{\mathrm{uc}} \omega_{ph}}} \epsilon_a \frac{\delta E_m(k)}{\delta u_a}. \quad (1)$$

for the Raman active modes and the electronic states at the K point the Brillouin zone. H^i_{ep} is the electron-phonon interaction Hamiltonian. k is the wavevector and m/m' are the quantum numbers of the intermediate electronic states. M_a denotes the atomic mass and N_{uc} the number of atoms in the unit cells. ϵ_a is the polarisation vector of atom a for a given phonon eigenvector, $E_m(k)$ the electronic eigenstate, and u_a the atomic displacement. The last term, $\delta E_m(k)/\delta u_a$, describes the change in electronic eigenenergy due to the displacement of the atoms according to a given phonon mode. We displaced (with phonopy) the a-th atom according to the eigenvector by $u_a = A\epsilon_a/\sqrt{N_{\mathrm{uc}} M_a}$, where $A = \pm 2, \pm 1, \pm 0.5$ Å is the amplitude (with masses given in atomic mass units). The energies of the electronic states at K were fitted under displacement with a linear function

to obtain $\delta E_m(k)/\delta u_a$ to extract the optical deformation potential following Refs. [18] and [19]. To converge the optical deformation potentials, we increased the k-point grid to $33 \times 33 \times 1$ points and ensured that the unoccupied states are converged with the same accuracy as the occupied states.

FITTING PROCEDURE FOR RAMAN SPECTRA

In order to plot a resonant Raman curve, a Raman spectrum like the one featured in Fig.1 was measured in a spectral range between 1.5 eV to 1.8 eV in steps of 5 nm at room temperature. A curve fitting program known as peak-o-mat was used to fit each Raman spectrum, and from the fits of the $MoSe_2$ A_{1g} mode and the WSe_2 E_{2g}/A_{1g} mode, we were able to extract the integrated intensity. These integrated peak intensities were then plotted with respect to excitation energy for both A_{1g} modes making resonant Raman curves featured in Fig.4(c,d) of the main text. This procedure was also done for the two individual monolayers and is featured as Fig 4(a,b).

The fits of the resonant Raman curves, seen as solid lines in Fig.4 of the main text, were done using Eq. 1 of the main text,

$$I_R(\omega_{ph}, E_l) \propto \sum_i \left| \frac{M_i^2 \cdot M_{ep,i}}{(E_l - E_i + i\gamma_i)(E_l - \hbar\omega_{ph} - E_i)} \right|^2, \qquad (2)$$

where M_i is the electric dipole matrix element of transition i and $M_{ep,i}$ its electron-phonon matrix element. E_l is the excitation laser energy, ω_{ph} the phonon frequency, and γ_i the decay rate of the excited electronic state. For the monolayers, and the WSe_2 A_{1g} mode, this equation was used in *Mathematica* for fitting unknown variables like the decay rates and the matrix elements with the use of known variables like the phonon energy and the optical transition energies. For Fig. 4(c), the $MoSe_2$ A_{1g} mode in the HT, it wasn't possible to fit it with an unaltered Eq. 1, but it is possible to fit if you consider the resonance curve in Fig. 4(c) to have two scattering pathways, one pathway the same as it is in monolayer, and the other pathway can be fitted with an additional identical term to Eq. 1 but with

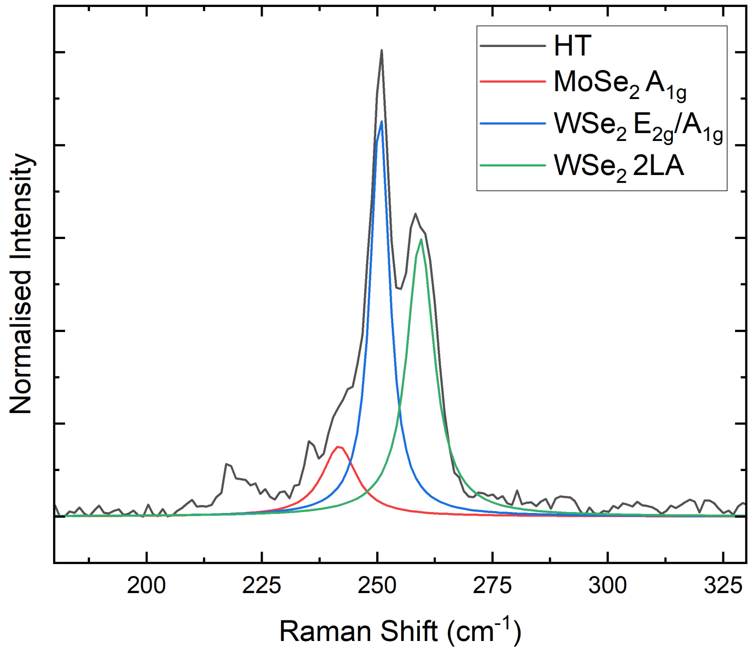

FIG. 1. Raman spectrum of a $MoSe_2/WSe_2$ heterostructure, with the A_{1g} modes of $MoSe_2$ at $242\,cm^{-1}$ (red), WSe_2 at $250\,cm^{-1}$ (also E_{2g} mode as they are degenerate), and a longitudinal acoustic phonon from WSe_2 at $261\,cm^{-1}$.

the transition energy of the WSe_2 A_{1g} mode.

$$I_R(\omega_{ph}, E_l) \propto \sum_i \left(\left| \frac{M_i^2 \cdot M_{ep,i}}{(E_l - E_i + i\gamma_i)(E_l - \hbar\omega_{ph} - E_i)} \right| + \ldots \left| \frac{M_i^2 \cdot M_{ep,i}}{(E_l - E_i + i\gamma_i)(E_l - \hbar\omega_{ph} - E_i)} \right| \right)^2 \tag{3}$$

Accounting for two scattering pathways instead of one made a convincing fit, and the fact that this second pathway has the same electronic transition energy as the *A* exciton in WSe_2, it suggests that the $MoSe_2$ A_{1g} mode can scatter from the intralayer excitons of both monolayers.

SUPPORTING FIGURES

Fig. 1(a) shows an AFM topography image, and (b) shows a scanning near field optical microscopy phase image (third harmonic phase image divided by the second harmonic phase image - ϕ_3/ϕ_2) of the $MoSe_2/WSe_2$ heterostructure. A scanning near field optical microscope (SNOM) is an optical microscope with the spatial resolution down to the

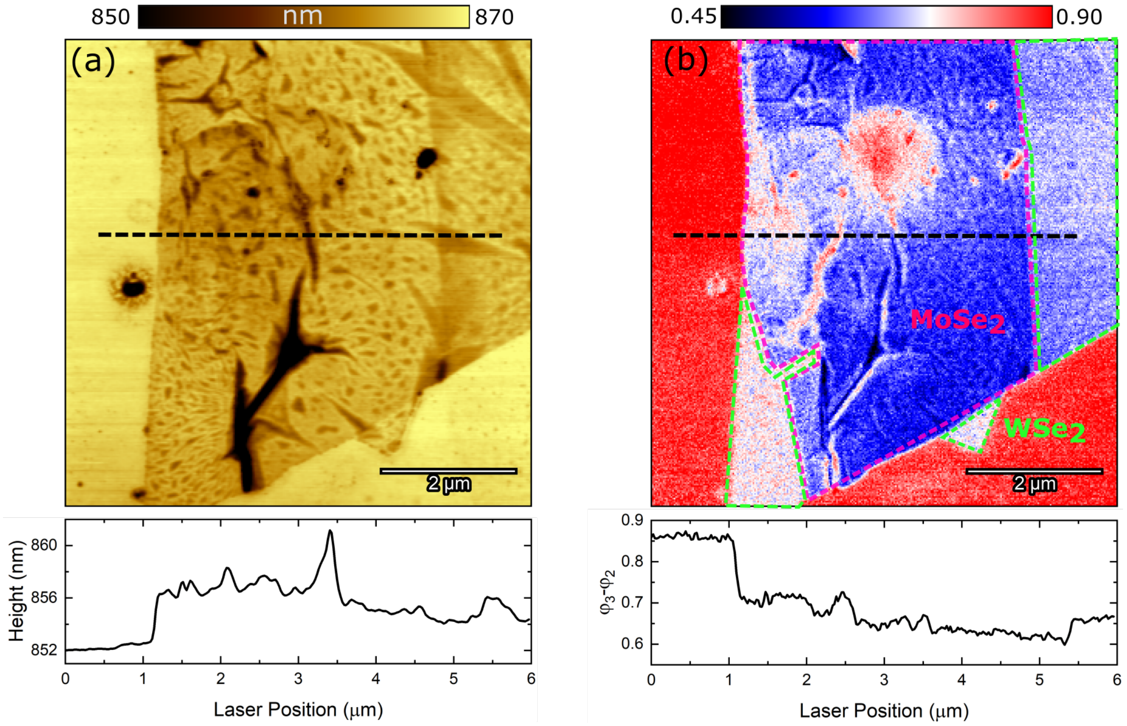

FIG. 2. (a) AFM topography image, (b) an optical phase image taken at 1.35 eV with a scanning near field optical microscope clearly showing the two monolayers through differing contrast values. The black dashed line indicates the line scan that is featured at the bottom.

nanometre range. Measuring with a SNOM yields an optical amplitude and phase image. SNOM measurements are sensitive to the dielectric function of the sample, the amplitude being sensitive to the real part of the dielectric function, the phase being sensitive to the imaginary part. The phase image in Fig. 1(b) was taken with an incident laser energy of 1.35 eV, and shows areas of different contrast values, indicating areas that possess different levels of optical absorption, making it easy to distinguish the monolayers making up the HT, with $MoSe_2$ in pink and WSe_2 in green.

Fig. 3 shows the PL from the individual monolayers of $MoSe_2$ and WSe_2, where it's clear to see the effect of a larger absorption coefficient for WSe_2 in comparison to $MoSe_2$.

Table I shows the full results for the deformation potential calculation of the main text. The letters is nomenclature taken from Ref. [20] and refers to layer orientation: H represents a rotation of 60° degrees with respect to the other layer, and R representing no rotation. The middle letter indicates a high-symmetry point in $MoSe_2$ (always h for hole) and the rightmost letter indicates which high-symmetry point of WSe_2 is above this point (h

	$M_{A_{1g}^M/XA^\circ}$ (eV/Å)	$M_{A_{1g}^W/XA^\circ}$ (eV/Å)	Stacking
$M_{A_{1g}^\circ/XA^M}$	104	-22	Hhh
	134	-30	RhX
	103	-22	RhM
	112	-29	HhX
	132	-25	HhM
	160	-28	Rhh
$M_{A_{1g}^\circ/XA^W}$	4	194	Hhh
	15	140	RhX
	9	78	RhM
	11	89	HhX
	19	200	HhM
	17	199	Rhh
	$M_{E_{2g}^M/XA^\circ}$ (eV/Å)	$M_{E_{2g}^W/XA^\circ}$ (eV/Å)	Stacking
$M_{E_{2g}^\circ/XA^M}$	0.000	0.003	Hhh
	0.008	-0.006	RhX
	0.005	0.002	RhM
	-0.007	-0.007	HhX
	0.004	-0.004	HhM
	0.000	-0.001	Rhh
$M_{E_{2g}^\circ/XA^W}$	0.001	-0.002	Hhh
	0.002	0.000	RhX
	0.000	0.001	RhM
	0.002	0.000	HhX
	0.001	0.000	HhM
	0.000	0.000	Rhh

TABLE I. Values determined for the deformation potential for the A_{1g}^M and A_{1g}^W modes (top) and for the E_{2g}^M and E_{2g}^W modes (bottom) on both the XA-excitons in $MoSe_2$ and WSe_2 bands at K. The circle (∘) indicates either M for $MoSe_2$ or W or WSe_2.

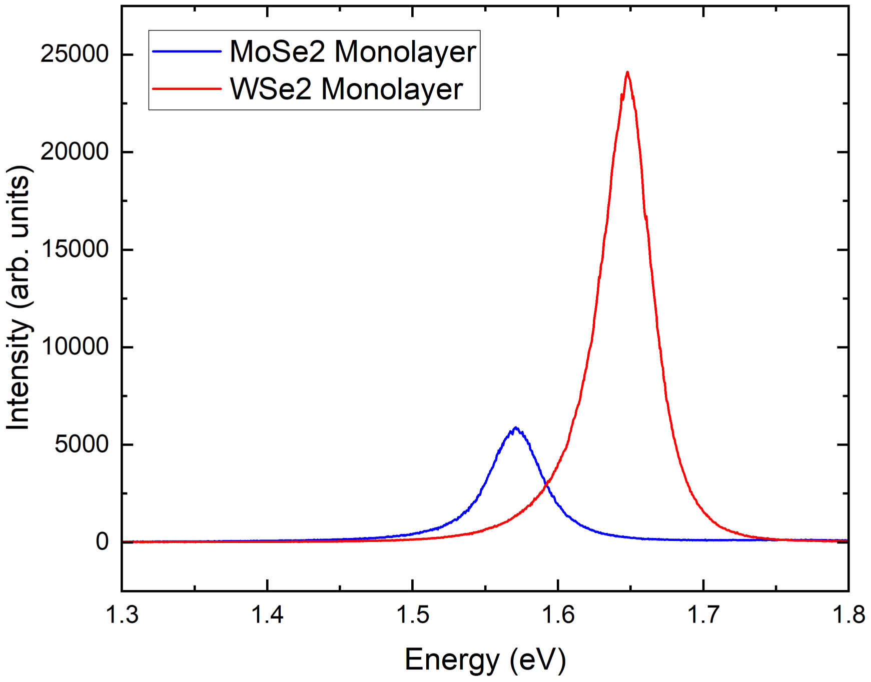

FIG. 3. Photoluminescence from the $MoSe_2/WSe_2$ monolayers, peak positions are 1.57 eV for $MoSe_2$ and 1.64 eV for WSe_2.

TABLE II. The position of the atoms and the relative displacement according to the Raman modes A_{1g}^{M} and A_{1g}^{W} used in the DFT calculation to obtain optical deformation potentials in Table II.

Element	Position (x,y,z)	A_{1g}^{M}	A_{1g}^{W}
Se	($\frac{2}{3}$,$\frac{1}{3}$,0.040)	0.114	-0.698
W	($\frac{1}{3}$,$\frac{2}{3}$,0.082)	-0.011	-0.008
Se	($\frac{2}{3}$,$\frac{1}{3}$,0.125)	-0.108	0.698
Se	($\frac{1}{3}$,$\frac{2}{3}$,0.876)	-0.695	-0.109
Mo	($\frac{2}{3}$,$\frac{1}{3}$,0.918)	0.003	0.008
Se	($\frac{1}{3}$,$\frac{2}{3}$,0.960)	0.702	0.113

for hole, M for the metal atom, i.e., X for the chalcogen, i.e., Se). Table II shows the details of the phonon eigenvectors used in the deformation potential calculation, the atomic positions, as well as the relative displacement according to the two Raman modes A_{1g}^{M} and A_{1g}^{W}. The negative(positive) sign on the displacements indicate downward(upward) move-

ment.

---

[1] A. Castellanos-Gomez, M. Buscema, R. Molenaar, V. Singh, L. Janssen, H. S. Van Der Zant, and G. A. Steele, Deterministic transfer of two-dimensional materials by all-dry viscoelastic stamping, 2D Materials **1**, 10.1088/2053-1583/1/1/011002 (2014).

[2] O. Garrity, A. Rodriguez, N. S. Mueller, O. Frank, and P. Kusch, Probing the local dielectric function of WS2 on an Au substrate by near field optical microscopy operating in the visible spectral range, Applied Surface Science **574**, 151672 (2022).

[3] M. Velický, A. Rodriguez, M. Bouša, A. V. Krayev, M. Vondráček, J. Honolka, M. Ahmadi, G. E. Donnelly, F. Huang, H. D. Abruña, K. S. Novoselov, and O. Frank, Strain and Charge Doping Fingerprints of the Strong Interaction between Monolayer MoS2and Gold, Journal of Physical Chemistry Letters **11**, 6112 (2020).

[4] P. K. Nayak, Y. Horbatenko, S. Ahn, G. Kim, J. U. Lee, K. Y. Ma, A. R. Jang, H. Lim, D. Kim, S. Ryu, H. Cheong, N. Park, and H. S. Shin, Probing Evolution of Twist-Angle-Dependent Interlayer Excitons in MoSe2/WSe2 van der Waals Heterostructures, ACS Nano **11**, 4041 (2017).

[5] P. Giannozzi, S. Baroni, N. Bonini, M. Calandra, R. Car, C. Cavazzoni, D. Ceresoli, G. L. Chiarotti, M. Cococcioni, I. Dabo, A. Dal Corso, S. De Gironcoli, S. Fabris, G. Fratesi, R. Gebauer, U. Gerstmann, C. Gougoussis, A. Kokalj, M. Lazzeri, L. Martin-Samos, N. Marzari, F. Mauri, R. Mazzarello, S. Paolini, A. Pasquarello, L. Paulatto, C. Sbraccia, S. Scandolo, G. Sclauzero, A. P. Seitsonen, A. Smogunov, P. Umari, and R. M. Wentzcovitch, QUANTUM ESPRESSO: A modular and open-source software project for quantum simulations of materials, Journal of Physics Condensed Matter **21**, 395502 (2009).

[6] P. Giannozzi, O. Andreussi, T. Brumme, O. Bunau, M. Buongiorno Nardelli, M. Calandra, R. Car, C. Cavazzoni, D. Ceresoli, M. Cococcioni, N. Colonna, I. Carnimeo, A. Dal Corso, S. De Gironcoli, P. Delugas, R. A. Distasio, A. Ferretti, A. Floris, G. Fratesi, G. Fugallo, R. Gebauer, U. Gerstmann, F. Giustino, T. Gorni, J. Jia, M. Kawamura, H. Y. Ko, A. Kokalj, E. Kücükbenli, M. Lazzeri, M. Marsili, N. Marzari, F. Mauri, N. L. Nguyen, H. V. Nguyen, A. Otero-De-La-Roza, L. Paulatto, S. Poncé, D. Rocca, R. Sabatini, B. Santra, M. Schlipf, A. P. Seitsonen, A. Smogunov, I. Timrov, T. Thonhauser, P. Umari,

N. Vast, X. Wu, and S. Baroni, Advanced capabilities for materials modelling with Quantum ESPRESSO, Journal of Physics Condensed Matter **29**, 465901 (2017).

[7] P. E. Blöchl, Projector augmented-wave method, Physical Review B **50**, 17953 (1994).

[8] A. Dal Corso, Pseudopotentials periodic table: From H to Pu, Computational Materials Science **95**, 337 (2014).

[9] J. P. Perdew, K. Burke, and M. Ernzerhof, Generalized Gradient Approximation Made Simple, Physical Review Letters **77**, 3865 (1996).

[10] S. Grimme, J. Antony, S. Ehrlich, and H. Krieg, A consistent and accurate ab initio parametrization of density functional dispersion correction (DFT-D) for the 94 elements H-Pu, Journal of Chemical Physics **132**, 10.1063/1.3382344 (2010).

[11] H. J. Monkhorst and J. D. Pack, Special points for Brillouin-zone integrations, Physical Review B **13**, 5188 (1976).

[12] T. Sohier, M. Calandra, and F. Mauri, Density functional perturbation theory for gated two-dimensional heterostructures: Theoretical developments and application to flexural phonons in graphene, Physical Review B **96**, 075448 (2017).

[13] A. Togo, First-principles Phonon Calculations with Phonopy and Phono3py, Journal of the Physical Society of Japan **92**, 10.7566/JPSJ.92.012001 (2023).

[14] A. Togo, L. Chaput, T. Tadano, and I. Tanaka, Implementation strategies in phonopy and phono3py, Journal of Physics: Condensed Matter **35**, 353001 (2023).

[15] J. Bardeen and W. Shockley, Deformation Potentials and Mobilities in Non-Polar Crystals, Physical Review **80**, 72 (1950).

[16] Z. Li, P. Graziosi, and N. Neophytou, Deformation potential extraction and computationally efficient mobility calculations in silicon from first principles, Physical Review B **104**, 195201 (2021).

[17] J. Maultzsch, H. Telg, S. Reich, and C. Thomsen, Radial breathing mode of single-walled carbon nanotubes: Optical transition energies and chiral-index assignment, Physical Review B - Condensed Matter and Materials Physics **72**, 10.1103/PhysRevB.72.205438 (2005).

[18] M. Machón, S. Reich, and C. Thomsen, Electron-phonon coupling in carbon nanotubes, in *Physica Status Solidi (B) Basic Research*, Vol. 243 (John Wiley & Sons, Ltd, 2006) pp. 3166–3170.

[19] F. S. Khan and P. B. Allen, Deformation potentials and electron-phonon scattering: Two

new theorems, Physical Review B **29**, 3341 (1984).

[20] H. Yu, G. B. Liu, and W. Yao, Brightened spin-triplet interlayer excitons and optical selection rules in van der Waals heterobilayers, 2D Materials **5**, 035021 (2018).
